# Label-Free Cytometric Evaluation of Mitosis via Stimulated Raman Scattering Microscopy and Spectral Phasor Analysis

**DOI:** 10.1021/acs.analchem.3c00212

**Published:** 2023-04-25

**Authors:** Ewan W. Hislop, William J. Tipping, Karen Faulds, Duncan Graham

**Affiliations:** Centre for Molecular Nanometrology, Department of Pure and Applied Chemistry, Technology and Innovation Centre, University of Strathclyde, Glasgow G1 1RD, United Kingdom

## Abstract

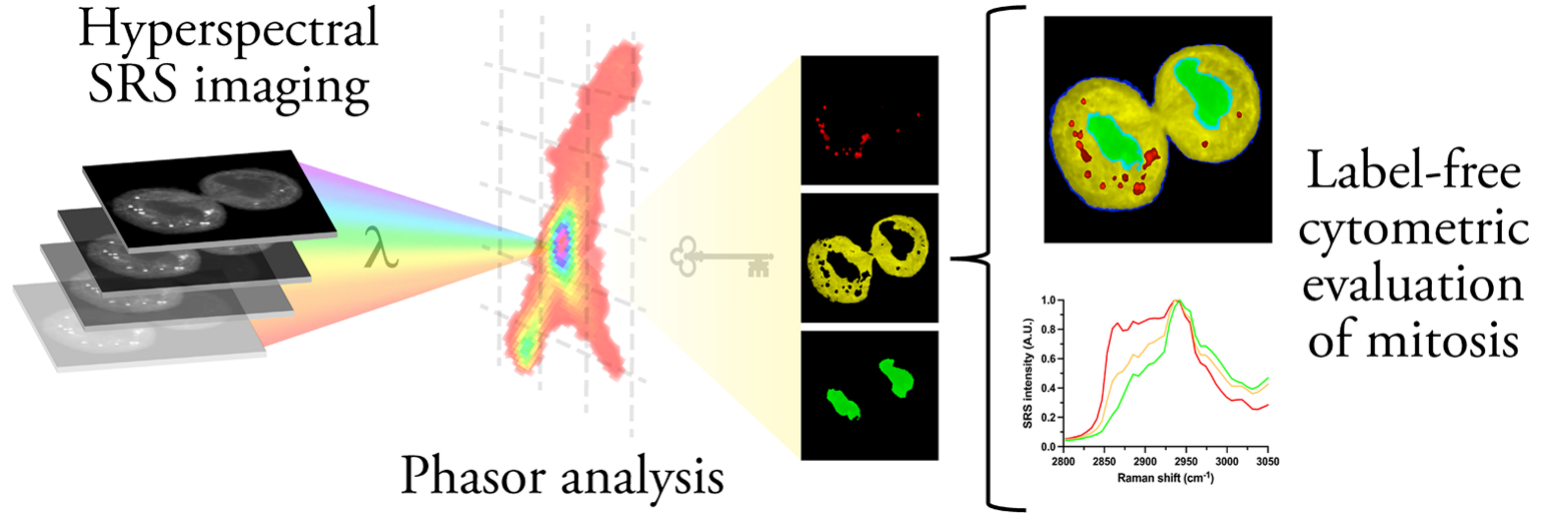

Hyperspectral stimulated Raman scattering (SRS) microscopy is a robust imaging tool for the analysis of biological systems. Here, we present a unique perspective, a label-free spatiotemporal map of mitosis, by integrating hyperspectral SRS microscopy with advanced chemometrics to assess the intrinsic biomolecular properties of an essential process of mammalian life. The application of spectral phasor analysis to multiwavelength SRS images in the high-wavenumber (HWN) region of the Raman spectrum enabled the segmentation of subcellular organelles based on innate SRS spectra. Traditional imaging of DNA is primarily reliant on using fluorescent probes or stains which can affect the biophysical properties of the cell. Here, we demonstrate the label-free visualization of nuclear dynamics during mitosis coupled with an evaluation of its spectral profile in a rapid and reproducible manner. These results provide a snapshot of the cell division cycle and chemical variability between intracellular compartments in single-cell models, which is central to understanding the molecular foundations of these fundamental biological processes. The evaluation of HWN images by phasor analysis also facilitated the differentiation between cells in separate phases of the cell cycle based solely on their nuclear SRS spectral signal, which offers an interesting label-free approach in combination with flow cytometry. Therefore, this study demonstrates that SRS microscopy combined with spectral phasor analysis is a valuable method for detailed optical fingerprinting at the subcellular level.

The mammalian cell cycle is regulated by a well-studied but complex biochemical reaction system, which has fascinated microscopists for over a century.^1^ Mitosis is the most distinctive phase of the cell cycle, which ensures genome integrity by orchestrating the precise segregation of the duplicated genetic material. In addition, the coordinated division of subcellular organelles during mitosis supports their inheritance and cellular homeostasis.^2^ Mitosis comprises five sequential stages known as prophase, prometaphase, metaphase, anaphase, and telophase/cytokinesis, where mechanisms such as chromatin condensation, nuclear envelope remodeling, modulation of the spindle apparatus, and chromosome alignment/separation facilitate equal distribution to the daughter cells with elegant precision.^2^ The functional dissection of mitotic biochemistry is still an active area of investigation due to the multifaceted nature of cell cycle dynamics and is of great importance to researchers, as its dysregulation has catastrophic consequences, including cancer.^3,4^ The development of approaches sensitive enough to evaluate the molecular profiles of individual cells provides an opportunity to gain a new perspective on some of the most fundamental processes of life.

Optical microscopy methods are often used to characterize cellular processes because they offer information on the distribution and dynamics of biomolecules at the single-cell level. Fluorescence microscopy is classically used for visualizing the specific structures and functions of biological systems;^5^ however, labeling target molecules is a key element to its execution. Labeling probes, such as fluorescent proteins or organic dyes, may perturb the intrinsic biochemical properties of the cell, and their application can often impair or modify its biological functions, coupled with potential imaging artifacts from nonspecific targeting.^6,7^ In addition, photobleaching, phototoxicity, and low multiplexing levels resulting from broad fluorescent spectra are also limitations of this method.^8^ Thus, label-free imaging modalities present a distinct advantage over the use of imaging dyes or fluorophores for visualizing cellular processes, i.e., the preservation of chemical specificity. Vibrational techniques like infrared (IR) spectroscopy can be used in histological analysis without the requirement of labels;^9,10^ however, IR radiation is strongly absorbed in aqueous specimens, and the resolution is restricted to whole-cell analysis.^11^ When imaging cellular models, Raman spectroscopy is a preferred optical tool because it can provide label-free characterization and distribution of biomolecules under biocompatible imaging conditions. This noninvasive technique has been widely applied to the study of cellular processes pertinent to biomedicine^12^ including cancer diagnostics,^13^ cardiovascular dysfunction,^14^ endothelial pathologies,^15^ and pharmacokinetics;^16^ however, long image acquisition rates can affect its biological sampling potential.

The development of stimulated Raman scattering (SRS) microscopy is credited with improvements in the spatial resolution, temporal analysis, and 3D imaging capabilities for bioanalysis using Raman spectroscopy.^17^ SRS has enabled the biochemical profiles of lipids, proteins, and nucleic acids within numerous biological models to be visualized including cells,^18,19^ tissues,^20,21^ and animals.^22,23^ Considering its high molecular specificity, sensitivity, and imaging rates, SRS imaging has emerged as a unique tool for pharmaceutical discovery.^24^ Drug uptake and distribution can be observed in high resolution through the use of endogenous chemical bonds or small bio-orthogonal tags,^25^ permitting intracellular pharmacodynamics to be monitored in real time.^26^ To enhance the chemical sensitivity of the SRS data obtained, a hyperspectral imaging approach can be applied,^27^ such that each pixel of an acquired image contains an intensity value as a function wavenumber in the Raman spectrum by automatic retuning of the pump wavelength in between image frames. To delineate these often rich hyperspectral data sets, multivariate analysis techniques have previously been employed, including principal component analysis (PCA), independent component analysis (ICA), and multivariate curve resolution (MCR), to extract underlying biochemical information from cellular constituents (e.g., nuclei, LDs, and cytoplasm).^28−30^ Recently, the application of *k*-means cluster analysis (KMCA) to multiwavelength SRS images in the high-wavenumber region generated a visual interpretation of endogenous LD composition in prostate cell models following drug perturbation.^31^ An alternative chemometric analysis tool for Raman spectral data is spectral phasor analysis, which has proven advantageous for cellular and tissue segmentation,^32,33^ cytometry,^34^ exploring drug–cell interactions,^35^ and chemoresistance mechanisms.^36^ SRS microscopy coupled with advanced chemometrics provides a label-free means to explore critical aspects of cell biology.

Herein, we report the use of spectral phasor analysis to assess subcellular features associated with mitosis and its associated spectral profile by SRS microscopy. A label-free assessment of the high-wavenumber region (2800–3050 cm^–1^) probed biomolecules at each phase of mitosis in the SK-BR-3 cell model. Cellular segmentation by multivariate analysis enabled the biochemical distribution of lipids, protein, and DNA to be considered specific to endogenous features in a robust and reproducible manner. This approach also allowed cells in the same field of view to be characterized in separate phases of mitosis without labeling. These results demonstrate the potential of hyperspectral SRS imaging and spectral phasor analysis for investigating fundamental processes of cell biology.

## Results and Discussion

The aim of this research was to capture the dynamic biological phases of mitosis and evaluate the molecular contributions which facilitate cell division via a label-free approach. Herein, we report the first application of hyperspectral SRS imaging, supplemented with spectral phasor analysis, to investigate the distribution of cellular DNA, protein, and lipids throughout mitosis. With readily identifiable and compartmentalized morphological structures, the SK-BR-3 cell model was considered an excellent candidate for phenotypic assessment. To probe its cellular biochemistry, a wavelength scanning experiment was performed by tuning the pump laser wavelength at increments of 0.4 nm (∼6 cm^–1^) across the range 2800–3050 cm^–1^ to collect a hyperspectral stack of 40 SRS images. A spectral phasor algorithm^32^ was applied to this data set, clustering each data point (spectral phasor) based on its SRS spectral similarity within the stack of images to generate a 2D chemical map of the cell model. Spectral phasor uses the Fourier transform to depict the spectrum of every pixel in a 3D hyperspectral SRS image stack as a point on the 2D phasor plane, providing a global overview of the ensemble of pixels. As such, spectral phasor analysis can simplify the interpretation of hyperspectral data sets to visualize the biochemical differences between cellular populations. Segmentation of the phasor plot enabled the identification of intracellular regions of interest (ROIs), and the corresponding SRS spectrum associated with the nuclear region could be used to probe the changes in cellular biomolecules during cell division.

**Figure 1:**
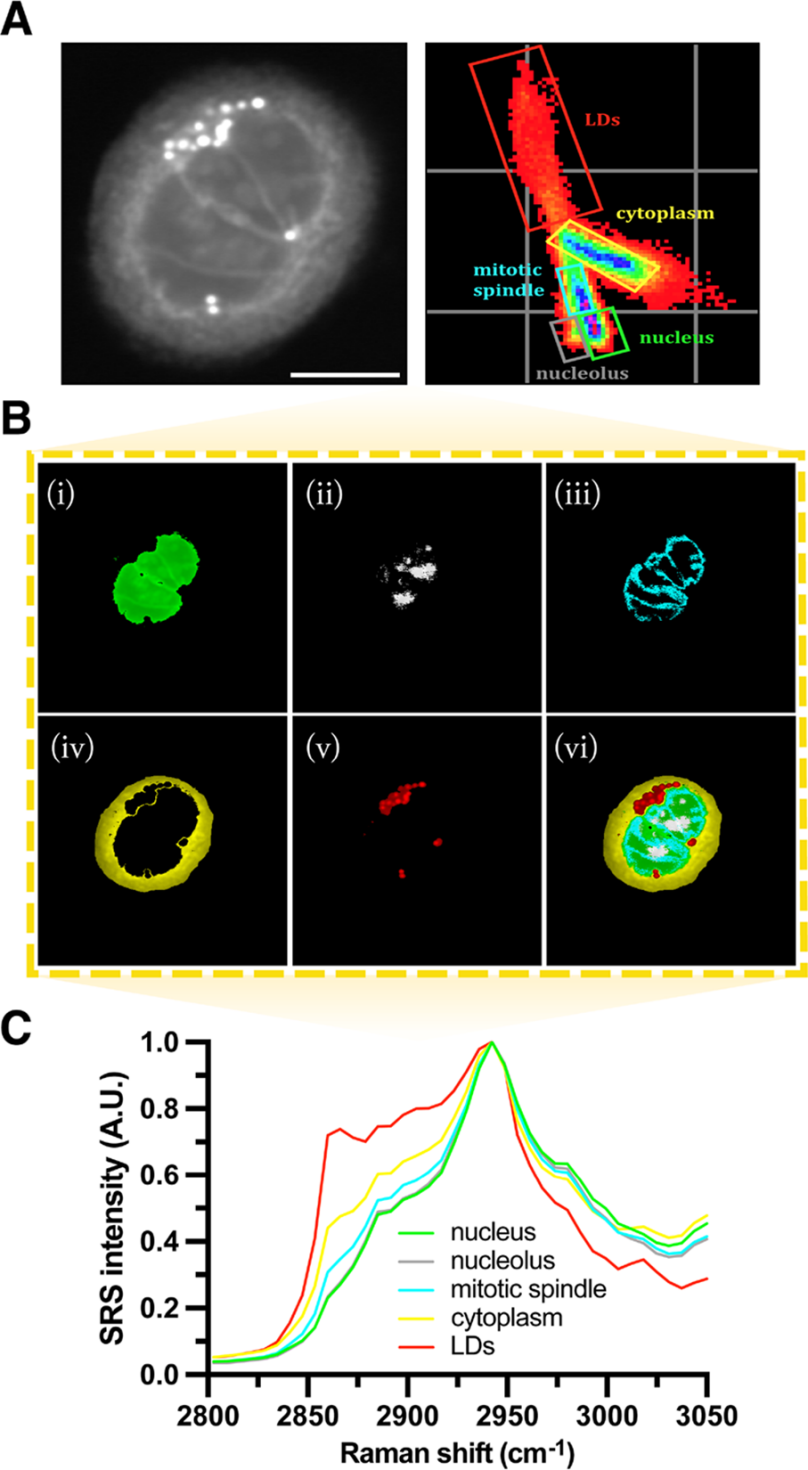
Spectral phasor segmentation of a SK-BR-3 cell in prometaphase. (A) Hyperspectral SRS images were attained across the high-wavenumber region (2800–3050 cm^–1^) and the application of phasor segmentation and analysis. An average intensity projection (scale bar 10 μm) of the image stack is presented alongside its spectral phasor plot. (B) The spectral phasor plot is segmented into five ROIs (i–v) associated with the biological properties of the cell: nucleus (i), nucleoli (ii), mitotic spindle (iii), cytoplasm (iv), lipid droplets (LDs) (v), and a composite image (vi). (C) Organelle-associated spectra were generated by phasor analysis and normalized between 0 and 1.

Phasor analysis of a single SK-BR-3 cell during prometaphase (Figure 1A) highlighted important biological structures within the cell including the nucleus (i), nucleoli (ii), mitotic spindle (iii), cytoplasm (iv), and lipid droplets (v). A composite image (iv) showcases the direct segmentation of all subcellular components (Figure 1B). Herein, label-free SRS imaging captures the fragmentation of the cell’s nuclear envelope, as evidenced by its morphology (i) and the initiation of mitotic spindle assembly (iii). During prometaphase, rapid growth of microtubules radiate out from the centrosomes to form a bipolar attachment with daughter chromosomes. This facilitates their movement toward the lateral poles (centrosomes) of the spindle in a highly dynamic fashion.^2^

Differentiation between cellular protein content is demonstrated in Figure 1B(i–iii) and outlines the dense histone-packed chromatin in the mitotic nucleus (i), the nucleolus (ii), and the microtubule formation of the spindle (iii). Phasor segmentation also allows the distribution and polarization of LDs during this mitotic event to be visualized (v). The close association between LDs and the spindle during mitosis has previously been described in NIH 3T3 cells, which may promote the uniform allocation of LDs to dividing daughter cells.^37^Figure 1C reports the unique organelle-associated spectra attained directly through phasor analysis and characterizes the biochemical distribution of molecules localized within these cell structures. Distinct spectral features of DNA (2970 cm^–1^), protein (CH_3_, 2930 cm^–1^), and lipids (CH_2_, 2851 cm^–1^) in the HWN region can be observed which are key C–H stretch assignments previously described in the DNA imaging of live cells.^18^ Lipids are predominately distributed throughout the cell cytoplasm and as LDs which serve a plethora of intracellular functions.^37^ In contrast, the total protein content of the cell is largest in the nucleus through its intrinsic association with DNA, where histones and many other nuclear proteins are localized.^2^ Interestingly, despite comparable spectral profiles (Figure 1C), the nucleoli are clustered independently from the rest of the nucleus (Figure 1A), which may reflect the different levels of DNA, RNA, and protein content across the nucleus as a whole. Our approach demonstrates that hyperspectral imaging of the HWN region (2800–3050 cm^–1^) in combination with phasor analysis offers a platform to explore the biochemical transformation of a cell during mitosis.

**Figure 2:**
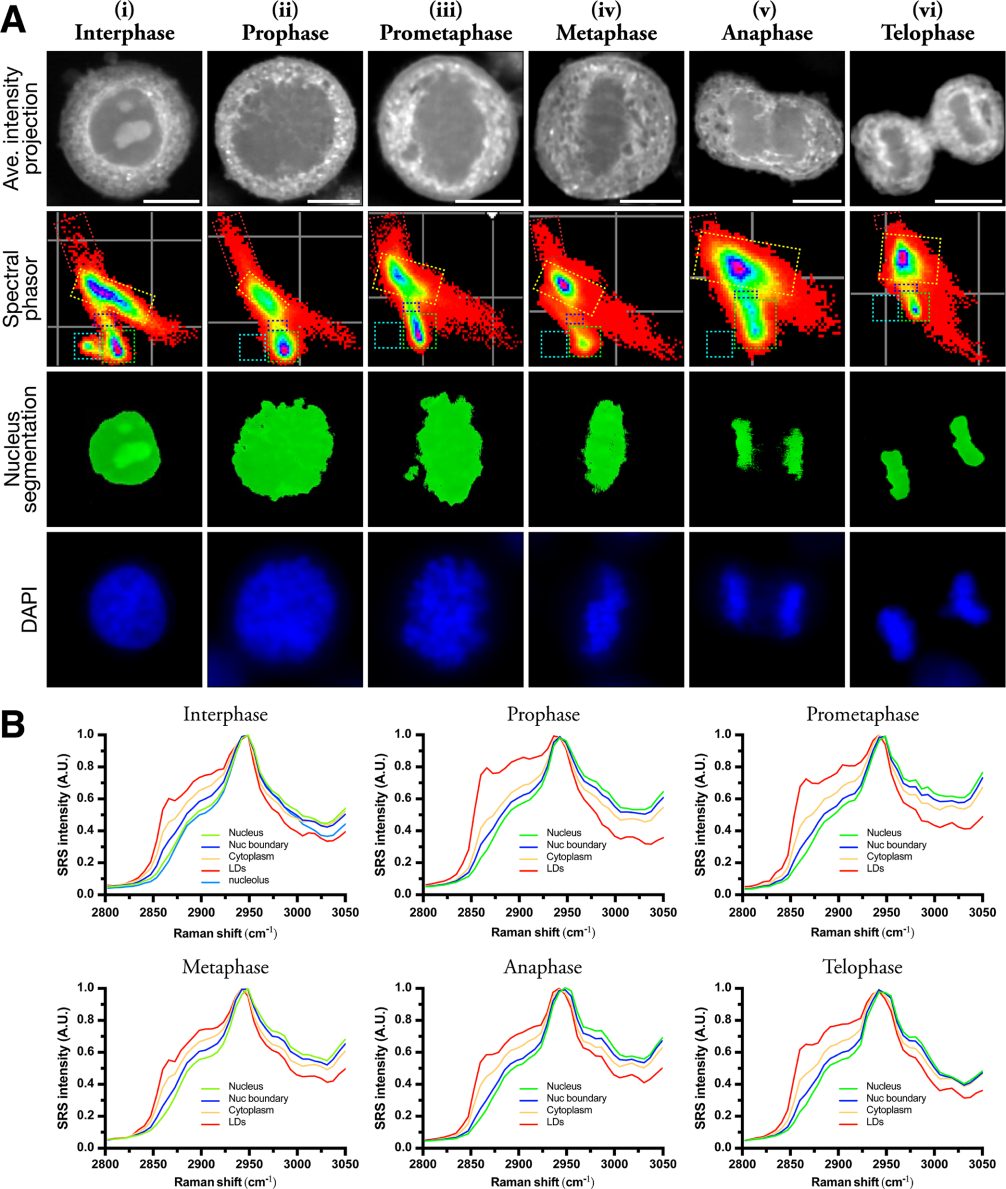
Spectral profiling of mitotic cells using phasor segmentation and analysis. (A) Three biological replicates were imaged at each of the five phases of mitosis and an interphase model for comparison (i–vi). SRS images were acquired across a range of wavenumbers (2800–3050 cm^–1^), and from this stack, an average intensity projection was generated (scale bar 10 μm). Spectral phasor plots were produced from each mitotic phase and segmentation of the nucleus. ROIs were assigned nucleus (green), nucleoli (cyan), nuclear boundary (blue), cytoplasm (yellow), and lipid droplets (red). Fluorescence imaging of DNA contrast agent (DAPI). (B) Organelle-associated spectra were created by phasor analysis (nucleus, nuclear boundary, cytoplasm, LDs, and nucleoli) and plotted in Graphpad, normalized between 0 and 1.

All phases of mitosis as well as the flanking periods of interphase and cytokinesis before and after are described by Alberts et al.^2^ To capture this short biological process (<1 h), we performed a double-thymidine block to synchronize a population of SK-BR-3 cells in culture and preserved the dividing cells in paraformaldehyde (PFA) at key time points during mitotic entry.^38^ Mitosis is a highly dynamic process, and under the microscope, live cells may become stressed; therefore, to minimize changes in cell biochemistry during the analysis, PFA fixation was chosen. Three biological replicates were imaged at each of the five phases of mitosis, and an interphase model was used for comparison. SRS images were acquired across a range of wavenumbers (2800–3050 cm^–1^), and from this stack, an average intensity projection was generated. Spectral phasor plots were then produced for each mitotic phase (as presented in Figure 2) and biological replicates (Figure S1A and S1B). To corroborate our label-free interpretation of the mitotic phases by SRS, we adopted a multimodal approach by labeling the cells with a DNA fluorescent dye (DAPI), which allowed sequential SRS microscopy and fluorescence imaging via a Leica microscope system. Parity between SRS imaging via phasor segmentation of the nucleus and the DNA contrast agent, DAPI, is evident, demonstrating the density and distribution of the condensed chromatin within the cell at each of the phases of mitosis (Figure 2A).

In early prophase, the homogeneously distributed chromatin of interphase form visible thread-like structures (Figure 2A(ii)), as it condenses into individual chromosomes to support their movement across the mitotic spindle without entanglement.^2^ In this compacted state, DNA can no longer be transcribed; therefore, all RNA synthesis ceases. Thus, at the end of prophase, as the site of RNA synthesis, the nucleolus disappears. The absence of the nucleolus during prophase correlates with its omission from the phasor plot (Figure 2A(ii)). While the complexity of nucleolar processing is an active area of research yet to be fully decoded, a comprehensive review^39^ illustrates the dynamics of its assembly/disassembly during the cell cycle. In HeLa cells, prophase is typically complete within 30 min and is reported in two separate phases: prophase a (proA) and prophase b (proB).^39^ A phasor approach readily segments the nuclear domain (Figure S2A) and delivers subtle molecular interactions specific to the nucleolus in proA and proB (Figure S2B), which may ultimately be overlooked during whole cell analysis. In the late prophase cell (proB), a noticeable change in SRS signal attained from the nucleolar segment is observed (Figure S2B(i)), while the ratio at 3020/2930 cm^–1^ associated with the localized RNA/protein levels^40^ declines (Figure S2B(ii)), concomitant with the ordered release of nucleolar complexes and repression of RNA transcription.^39^ These data suggest it is possible to identify chemical contrast within the mammalian nucleus at different cell cycle stages. In addition, multiple labels would be required to resolve these features by fluorescence imaging, whereas label-free SRS can characterize these endogenous structures efficiently and effectively.

**Figure 3:**
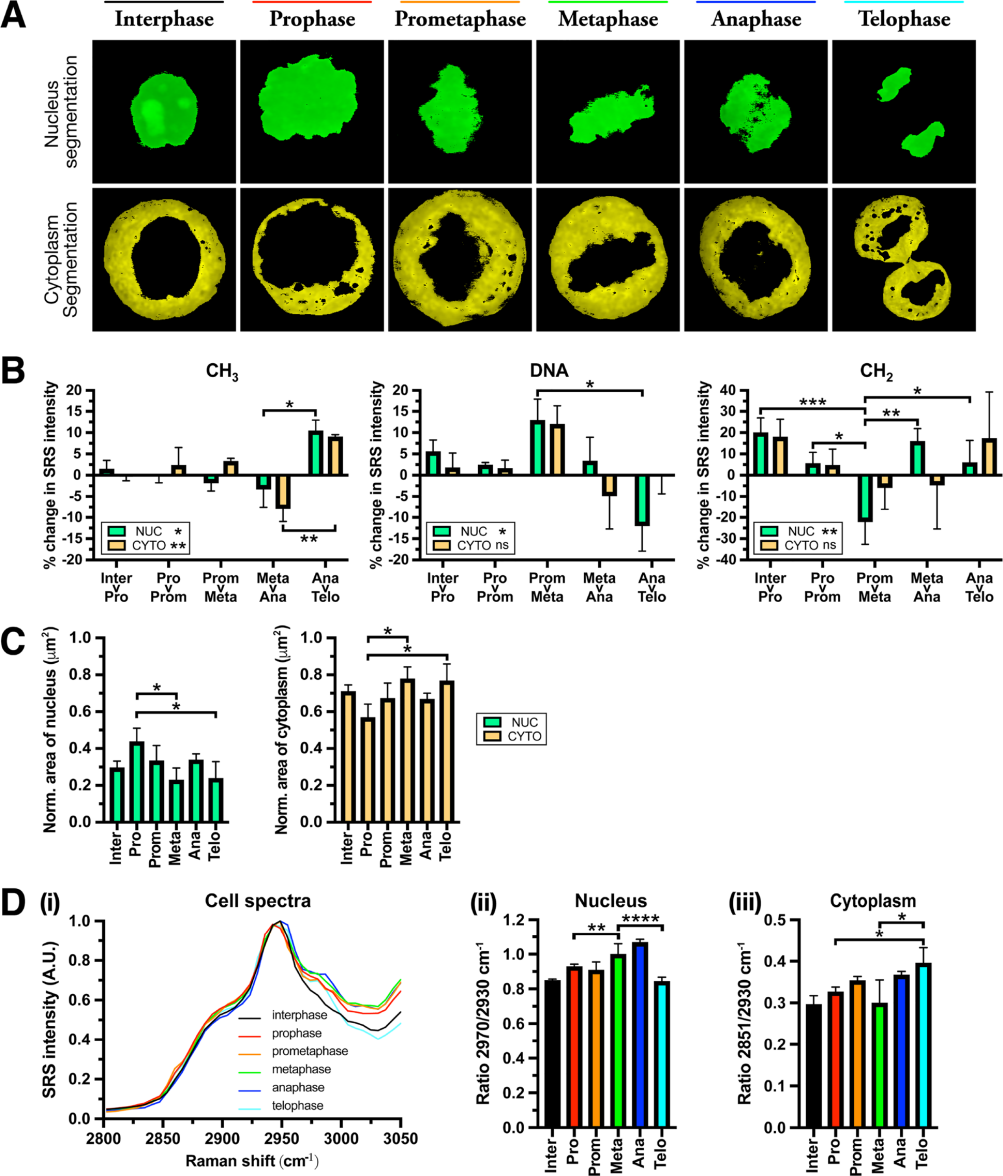
Phasor analysis of the nuclear and cytoplasmic compartments. (A) Segmentation of both compartments, nucleus (green) and cytoplasm (yellow). (B) The mean percentage change (%) in SRS intensities at 2930 (CH_3_), 2970 (DNA), and 2851 cm^–1^ (CH_2_) between sequential phases in premitotic and mitotic cells (interphase > telophase) at nuclear and cytoplasmic regions. (C) Quantification of the mean surface area (μm^2^) of nuclear and cytoplasmic compartments in the segmented image during mitotic events. Mean area of each compartment was normalized to the total area of the cell. (D) The average mitotic cell spectra from SRS images acquired across a range in the high-wavenumber region (2800–3050 cm^–1^) (i) and ratios at 2970/2930 cm^–1^ (DNA/CH_3_) from the nucleus and (ii) 2851/2930 cm^–1^ (CH_2_/CH_3_) in the cytoplasm (a.u.) (iii). Three replicate analyses were acquired for each cell cycle phase. Data represent the mean ratio ± SD. A one-way Anova test with Tukey posthoc analysis was performed. * *P* ≤ 0.05, ** *P* ≤ 0.01, **** *P* ≤ 0.0001.

The nuclear envelope (NE) in eukaryotic cells provide a physical barrier to separate DNA replication and transcription from other cytoplasmic events.^2^ Complete dissolution of the NE characterizes the beginning of prometaphase (Figure 2A(iii)) and facilitates the movement of molecules along their concentration gradients unrestricted by membranes.^41,42^ Thus, the nuclear and cytoplasmic environments become more homogeneous,^43,44^ as represented by the clustering of pixels on the phasor plot (Figure 2A(iii)) in comparison to prophase (Figure 2A(ii)). The average spectra extracted from the cytoplasm details a small increase in the intensity of the CH_2_ lipid shoulder peak at 2851 cm^–1^ with concomitant endogenous lipid remodeling (Figure 2B(iii)). However, the structural complexity of the NE and the speed at which it disassembles coupled with its nanoscale diameter (10–50 nm) prevent direct NE correlations from being drawn due to the resolution limit of the SRS system employed (∼400 nm). Nevertheless, phasor segmentation of the nucleus and cytoplasm did permit biochemical assessment, which supports the molecular reconfiguration in both compartments associated with NE dissolution (Figure 3A). The CH_2_ signal collected from both compartments shows a ∼5% change in intensity associated with lipids. In addition, at this stage, it is reported that nuclear proteins transition into the cytoplasm until late anaphase, where they are reimported into daughter nuclei shortly before segregation.^41^ Here, we observed a ∼2.5% change in the intensity of the CH_3_ signal in the cytoplasmic compartment, whereas no change was seen in the nucleus (Figure 3B).

Upon disintegration of the NE, microtubules of the spindle radiating out from the polar centrosomes can now access the chromatin, attach, and orchestrate migration until pole-directed forces are balanced.^2^ Once aligned at the equator of the spindle in metaphase, the chromatin adopts its most compacted state during mitosis (Figure 2A(iv)). Phasor segmentation allowed dimensional modifications to the cell during mitosis to be characterized, which demonstrates the nucleus had the lowest surface area in metaphase (Figure 3C). The corresponding phasor plots from cells in metaphase (Figure 2A(iv)) exhibit a reduction in the intensity of pixels associated with the nucleus, which may be a direct reflection of this nuclear compaction or the passage of proteins and lipids into the cytosol (Figure 3B). During the process of chromatin condensation, the local concentration of histones and DNA packaging proteins within the nucleus increases, which may induce a redistribution of proteins dissociated from their transient bonds to DNA, driven by a concentration gradient.^45^ The percentage change in SRS intensity in the nucleus (∼12.5%) between prometaphase and metaphase at 2970 cm^–1^ (DNA) may be indicative of this process (Figure 3B). In contrast, lower rates of condensation tend to occur in interphase, as heterochromatin (the condensed state of chromatin) represents only a part of the whole chromatin in the nucleus.^46^ This may explain the difference in the intensity ratio (DNA:CH_3_) values observed between mitotic and interphase cells (Figure 3D).

**Figure 4:**
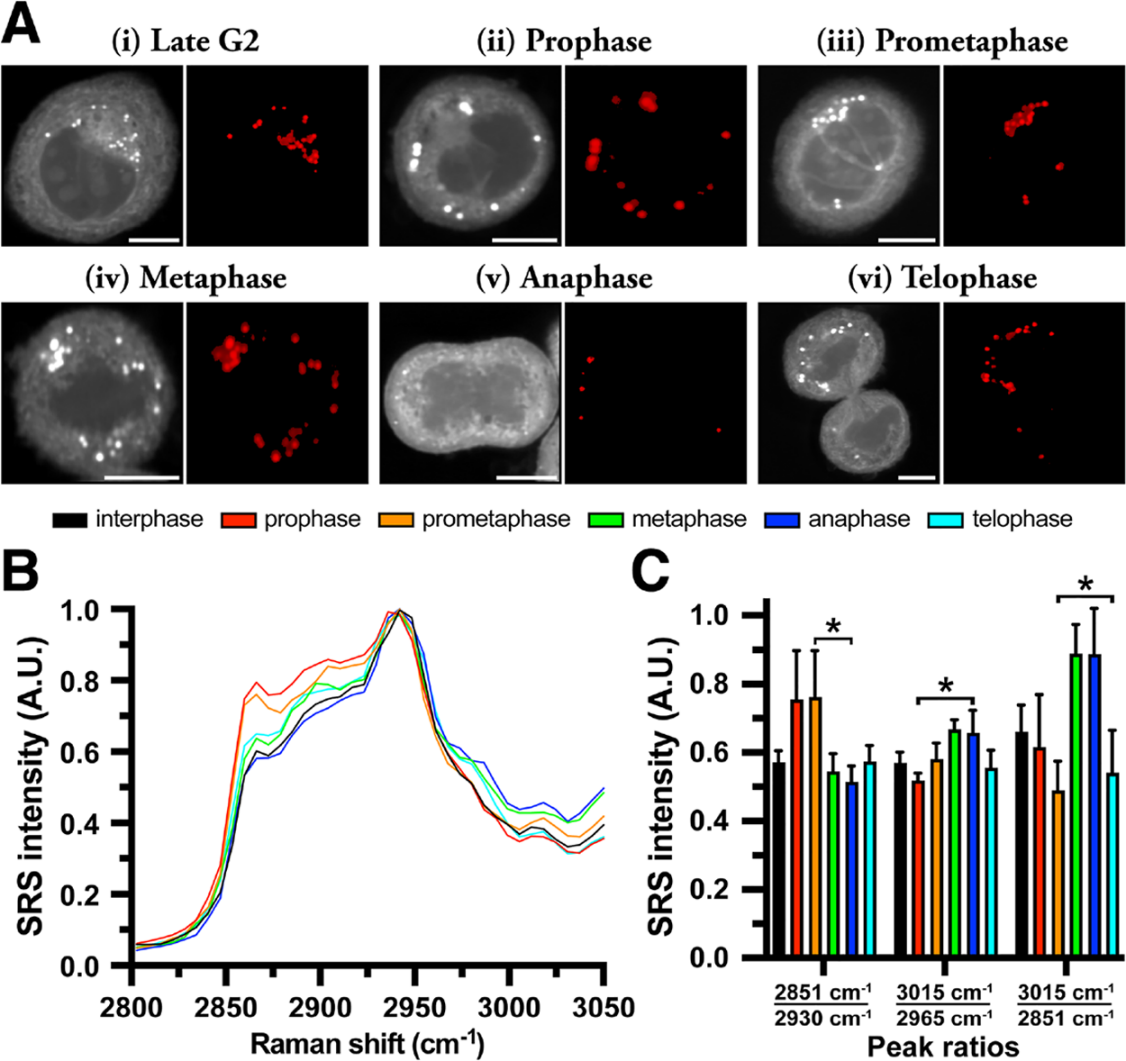
Visualizing the distribution and content of LDs during mitotic events by phasor analysis. (A) SK-BR-3 cell model at phases of mitosis, and the distribution of LDs. SRS images were collected across a range of wavenumbers (2800–3050 cm^–1^), and from this stack, an average intensity projection was generated (scale bar 10 μm). The corresponding segments from the phasor plots indicate the distribution of LDs during mitosis. (B) The average LD spectrum is plotted using the segmented LD image as a marker to determine the mean SRS spectrum at each mitotic phase. Representative peaks: 2851 (CH_2_ symmetric stretch), 2930 (CH_3_ symmetric stretch), 2965 (cholesterol esters, CEs), and 3015 cm^–1^ (=CH unsaturated lipid). (C) Ratiometric analysis of the peak intensities associated with LD-stored content at 2851/2930 (CH_2_/CH_3_), 3015/2965 (=CH/CEs), and 3015/2851 cm^–1^ (=CH/CH_2_). Three replicate analyses were acquired for each cell cycle phase. Data represent the mean ratio ± SD. A one-way Anova test with Tukey posthoc analysis was performed. * *P* ≤ 0.05.

The transition into anaphase is tightly regulated by a checkpoint mechanism, which ensures precise chromosome alignment and spindle integrity are retained.^2^ In anaphase, motor proteins (i.e., kinesin and dynein) that link the spindle microtubules facilitate elongation between the poles, prizing the homologous chromosomes apart toward opposite ends of the cell (Figure 2A(v)). On the representative phasor plots of anaphase, clustering between nuclear and cytoplasmic regions was unique. When selected, this highlighted the large abundance of spindle microtubules crucial for segregation. Subcellular structures also partition at this time, and the formation of a cleavage furrow marked the future site of abscission.^47^ Across the replicate images of mitosis, LD clusters were typically found adjacent to the nucleus and featured on the phasor plots; however, very few LDs were visualized during anaphase (Figures 2A(v) and S1). This finding led us to probe the LD distribution using phasor segmentation (Figure 4).

In mammalian cells there are only a limited number of studies on LD dynamics during mitosis; however, it is believed that the distribution of LDs changes from a dispersed state to a highly aggregated state in response to microtubule remodeling, as they are closely associated with the spindle.^48^ Herein, label-free SRS imaging captured LDs associated with two features which resemble centrosomes adjoined by spindle-like fibers during prophase (Figure 4A(ii)). In contrast, a cell imaged in late G2 phase displayed aggregation solely around one site where centrosome migration is yet to occur (Figure 4A(i)). In prometaphase, spindle microtubules now have direct access to the nuclear content; notable polarization of the LDs was observed (Figure 4A(iii)). Despite sparse records of this behavior during the mammalian cell cycle, Cruz *et al*. first reported symmetrical polarization of LDs when the cells approached metaphase in BODIPY-stained NIH-3T3 cells by fluorescence microscopy.^37^ The authors proposed that LD polarization allows the homogeneous distribution of LDs between mother and daughter cells; however, the study did not focus on later mitotic events. Here, we visualize LD dynamics throughout mitosis using a label-free approach and probe the variation of lipid biomolecules specific to the LDs in SK-BR-3 cells (Figure 4B and 4C). As a marker for assessing the composition of LDs, we calculated the ratios of the intensity between triacylglycerols (TAGs, 3015 cm^–1^, CH), cholesterol esters (CEs, 2965 cm^–1^), and saturated lipids (CH_2_, 2851 cm^–1^).^35,49^ An increase in saturated lipids was observed at early prophase and prometaphase in both LD spectra (Figure 4B) and the ratio between 2851/2930 cm^–1^ (Figure 4C). In contrast, during metaphase and anaphase, the ratios at 3015/2965 and 3015/2851 cm^–1^ are elevated in LDs, which suggests increased levels of unsaturation in stored neutral lipids.^49^ We postulate that compositional changes to these stored lipids may be associated with the destruction and reassembly of the nuclear membrane^50^ or the storage of building blocks to support membrane physiology following cytokinesis.^47^ In addition, a recent study in eukaryotic yeast models reported that the storage of unsaturated lipids in cytoplasmic LDs may buffer the nuclear envelope from structural abnormalities.^51^ Given that mitosis comprises many dynamic events of the membrane, it is feasible that LDs may play a role in the remodeling of membrane structure or generating lipid signaling molecules to govern the cell cycle transitions.^52^

As cells begin to exit division during telophase, microtubules which formed the spindle contract into an intercellular bridge (midbody), where abscission takes place (Figure 2A(vi)). Subsequently, restructured ER membranes enclose daughter nuclei to regenerate the nuclear envelope.^53^ Segregation between the nuclear and the cytoplasmic regions on the phasor plot may reflect the division of both components into new cells (Figure 2A(vi)). Nuclear proteins that were released into the cytoplasm during NE disassembly are reimported back to the newly formed nuclei (Figure 3B). Endogenous trafficking events such as endocytic recycling or plasma membrane remodeling are thought to contribute to the formation of new membranes during late mitosis; however, the identity and spatiotemporal properties of identified organelles during late cell division remain unclear.^54^ Phasor segmentation of both cell and nuclear boundaries were probed during this study to evaluate localized changes in areas which contain membranous structures (Figure S3). Due to the essential role lipid unsaturation plays in the biophysical properties of membranes (e.g., shape and fluidity),^55^ we analyzed the ratio at 3015/2851 cm^–1^ (total unsaturated/total saturated lipid).^56^ Overall, we observed higher rates of lipid unsaturation during mitosis in the cytoplasm (Figure S3A), cell boundary (Figure S3B), and nuclear boundary (Figure S3C), with the highest rates of unsaturation in membrane-associated regions (Figure S3C). The highest degree of unsaturation associated with membrane fluidity was found during anaphase (Figure S3B ans S3C), and where both the expansion of the plasma membrane aids correct spindle alignment for symmetric division and the nuclear membrane reassembly transpires.^57^ In addition, NE stability is influenced by the degree of saturation of lipids in ER membranes which could regulate the enzyme activity of lipids to control lipid content and abundance crucial to its reformation.^58,59^ The lowest measurements of unsaturation were recorded in telophase, where a decrease in membrane turnover is typically observed at this final stage of the cell cycle.^60^ Nuclear reassembly and chromatin decondensation re-establish the cells’ interphase structure, as the nucleolus reforms and transcription of RNA begins. In contrast to anaphase and metaphase, the ratio of DNA to protein (2970/2930 cm^–1^) decreased during telophase, since the distribution of DNA has already become relatively diffuse at this late stage of mitosis (Figure 3B and 3D). The intensity of the SRS signal associated with protein (CH_3_) indicates extensive redistribution in both nuclear and cytoplasmic compartments (Figure 3B). SRS imaging using a spectral phasor approach showcases chromatin distribution during mitosis (Figure 2), which parallels that observed via fluorescence (DAPI) staining and may provide a novel approach to analyze cell division. As a label-free technique, this analysis was attained without perturbing cellular biochemistry, whereas the visualization of immunofluorescent labels or organic dyes requires membrane permeabilization and multiple staining techniques.^7^ Single-cell phasor analysis enabled segmentation between spectral features of significance and their associated biological properties.

**Figure 5:**
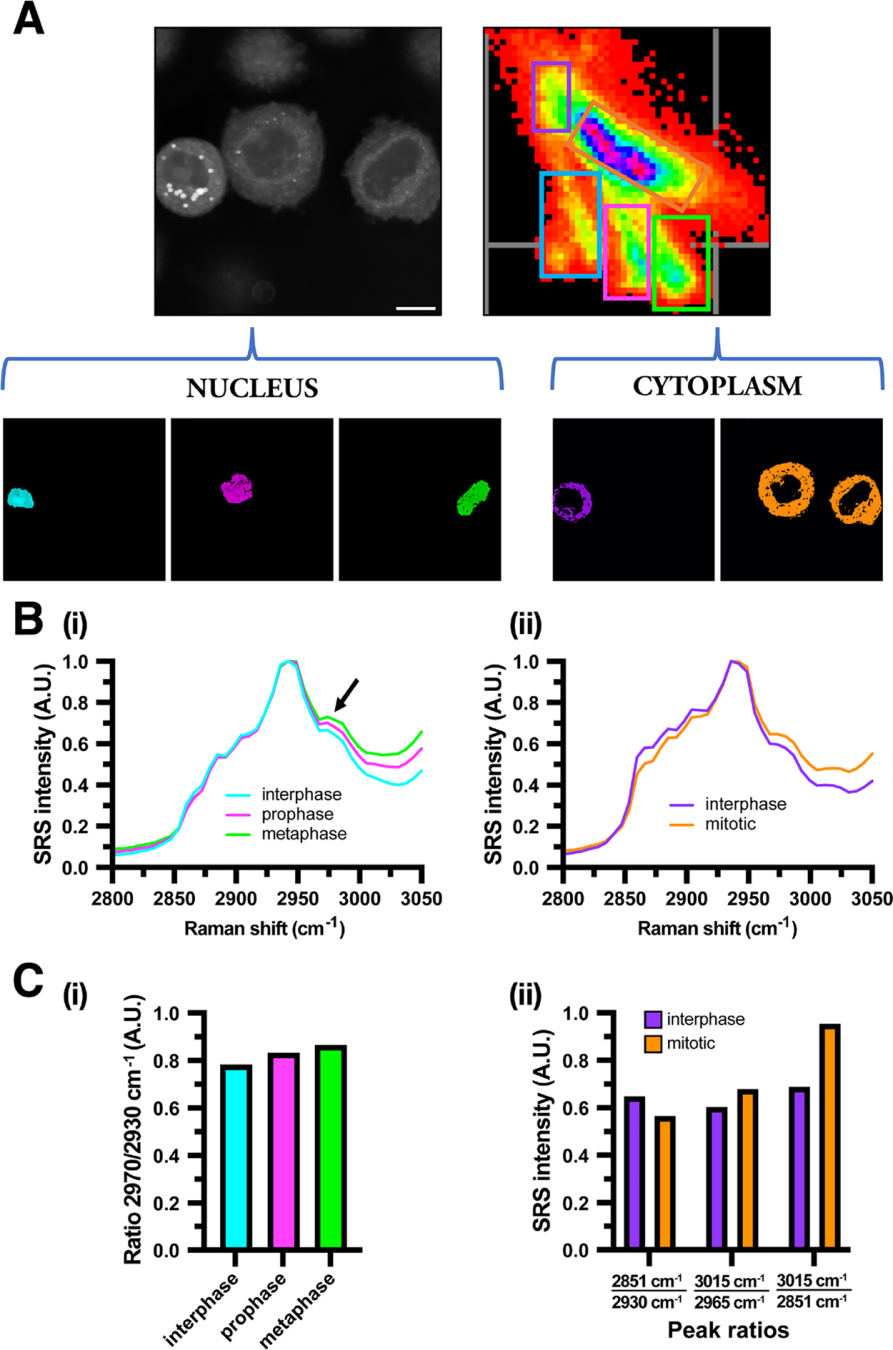
Cell cycle differentiation via a spectral phasor approach. (A) An average intensity projection of three mitotic cells (scale bar 10 μm), and the corresponding phasor plot was generated. Segmentation of the phasor plot indicated 3 ROIs associated with different nuclei, (i) interphase (cyan), prophase (magenta), metaphase (green), and cytoplasmic regions, (ii) mitotic (orange) and interphase (purple). (B) Spectra associated with the signal from the nucleus (i) and cytoplasmic (ii) regions of the SRS image were extracted by phasor analysis. The arrow indicates the DNA peak at 2970 cm^–1^. (C) Ratiometric analysis of the intensity at 2970/2930 cm^–1^ (DNA/CH_3_) between imaged nuclei (i), and intensities at 2851/2930,3015/2965 and 3015/2851 cm^–1^ of cytoplasmic regions (ii).

During our investigation we discovered that a spectral phasor approach can also be utilized to detect cells in different phases of division, as shown in the segmented SRS images presented in Figure 5A. A phasor plot of the hyperspectral stack of images presents three distinct areas of pixels clustered separately and correspond to individual nuclei. Closer visual inspection of each cell and its nucleus highlighted a cell in interphase (cyan), prophase (magenta), and metaphase (green). Spectral profiling of each nucleus showed variation in the DNA peak at 2970 cm^–1^ (Figure 5B), which may offer a novel label-free approach to cytometric analysis, particularly in combination with flow cytometry.^61^ Interestingly, this approach did not segment the cytoplasm of each cell independently; instead, we observed segregation between cells in mitosis and interphase (Figure 5B(ii)). Ratiometric analysis of key spectral assignments revealed variation between individual nuclei at 2970/2930 cm^–1^ (Figure 5C(i)). The compositional assessment of cytoplasmic LDs (Figure 5C(ii)) indicated a significant increase in the ratio value at 3015/2851 cm^–1^ between cells in mitosis and interphase. This result is comparable to our findings in Figure 4C and may indicate a greater storage of unsaturated lipids in mitotic cells preparing for extensive membrane remodeling in late mitosis.^47^ Traditional methods for cell cycle phase classification rely on the use of DNA-binding fluorescent dyes to inspect nuclear dynamics, which can be challenging due to nonspecific binding and dye-associated toxicities.^5^ Furthermore, labeling small molecules with bulky fluorophores typically changes their physical and biological properties, for example, the use of Nile Red as a hydrophobic stain for lipid droplets.^62^ In contrast, SRS microscopy examines molecular information specific to the cells’ biochemistry without labeling, and using a phasor-based approach facilitates spectral profiling of its endogenous components (e.g., the nucleus) as a function of its composition.^35^

## Conclusion

We have reported the use of hyperspectral SRS imaging to visualize the intrinsic vibrational signals from single cells to measure their biochemistry throughout mitosis. Label-free SRS imaging of this fundamental process of eukaryotic life captured the gradual spectral changes in biomolecules at various subcellular compartments. A spectral phasor approach to image analysis demonstrated chromatin distribution during mitosis, which paralleled the fluorescent DNA contrast agent without disturbing cellular composition. Our results also demonstrate the use of chemometric-based image analysis for cell cycle differentiation, which in tandem with flow cytometry could be a transformative platform for label-free cytometry. Potential future research may include the development of organelle-specific probes (e.g. bio-orthogonal Raman tags) that facilitate the precise imaging of division structures, which would enhance the spatial resolution of these dynamic nanoscale subcellular components during the cell cycle. In addition, automatic segmentation of the spectral phasor plot using machine learning methodology that is based on a priori spectral features (for example, the SRS spectrum of DNA) could increase the throughput of the technique, the reproducibility of the phasor segmentation, and the end-user accessibility. To conclude, SRS microscopy coupled with spectral phasor analysis offers a versatile modality to visualize the role of molecules at the subcellular level.

## Data Availability

The research data associated with this paper will become available from the University of Strathclyde at the following link: https://doi.org/10.15129/a391c7e3-25b9-4b30-88f8-7257f01b27bf.

## Supporting Information Available

Methodology for all of the experiments reported in the article, SRS images of biological repeats, assessment of the nucleolus and membrane-associated areas by spectral phasor analysis (PDF)

## Abbreviations

DAPI: 4′,6-diamidino-2-phenylindole
DNA: deoxyribonucleic acid
EOM: electro-optic modulator
FBS: heat-inactivated fetal bovine serum
LDs: lipid droplets
HWN: high-wavenumber region
NE: nuclear envelope
OPO: optical parametric oscillator
PBS: phosphate-buffered saline
PFA: paraformaldehyde
RNA: ribonucleic acid
RPMI: Rosewell Park Memorial Institute medium
ROIs: regions of interest
SK-BR-3: human breast cancer cell line
SRS: stimulated Raman scattering
TAGs: triacylglycerols.
