# Supplementary material for: Label-Free Cytometric Evaluation of Mitosis via Stimulated Raman Scattering Microscopy and Spectral Phasor Analysis
Source: Anal Chem. 2023 Apr 25;95(18):7244–53. doi: 10.1021/acs.analchem.3c00212 (PMC10173251; doi:10.1021/acs.analchem.3c00212)
Supplement: Supplementary file 1 — ac3c00212_si_001.pdf [file ac3c00212_si_001.pdf]

Supporting Information for

Label-free cytometric evaluation of mitosis via stimulated Raman scattering microscopy and spectral phasor analysis

Ewan W. Hislop, William J. Tipping, Karen Faulds,* and Duncan Graham*

Centre for Molecular Nanometrology, Department of Pure and Applied Chemistry, Technology and Innovation Centre, University of Strathclyde, 99 George Street, Glasgow, G1 1RD, U.K.

*Email: duncan.graham@strath.ac.uk; karen.faulds@strath.ac.uk

Table of Contents

1. Experimental Procedures *3*

1.1. Cell Culture and Synchronization 3

1.2 Stimulated Raman Scattering Microscopy 3

1.3 Fluorescence imaging 3

1.4 Data Processing 4

2. Supplementary figures *5*

Figure S1 Hyperspectral profiling of mitotic cells using phasor analysis 5

Figure S2 SRS imaging of nucleolar disassembly at the beginning of mitosis 6

Figure S3 Investigating membrane associated regions of mitotic cells by phasor analysis 7

3. References *8*

1. Experimental Procedures

1.1. Cell Culture and Synchronization

Cell Culture. SK-BR-3 cells (ATCC® HTB-30™) were obtained from American Type Culture Collection (ATCC). Live cells were cultured in Rosewell Park Memorial Institute medium (RPMI 1640; GIBCO™, Fisher Scientific) supplemented with 10% foetal bovine serum (FBS, Gibco™, Fisher Scientific), 1% penicillin/streptomycin (Gibco™, 10 000 U mL^{-1}, Fisher Scientific) and 1% amphotericin B (Gibco™, 250 μg mL^{-1}, Fisher Scientific). Cells were propagated in T-175 Falcon flasks at 37 °C within a humidified incubator containing 5% CO_2, where exponential growth was sustained between 0.25 and 1.5×10^6 cells/mL. Routine subculture was performed at ca. 80%. For SRS experiments, cell cultures were passaged and harvested every 3–4 days.

Cell Synchronization. Thymidine bioreagent was obtained from Sigma Aldrich (>99 %) and used as supplied. SK-BR-3 cells at ~30 % confluency were washed with PBS (2 × 2 mL) and grown in the presence of 2 mM thymidine for 18 h. After the first block, thymidine was removed, cells were washed and grown in fresh culture medium for 9 h to release cells from the block. Subsequently, a second block was applied by the addition of 2 mM thymidine and cultivation for a further 17 h. As a result of synchronization, cells progress synchronously through G2 and mitotic phases and will be arrested at the beginning of S phase. After release from the thymidine block, >95 % of the cells entered S phase (in 0–4 h), progressed into G2 phase (5–6 h), underwent a synchronous mitosis at 7–8 h, as described by Banfalvi.[1]

1.2 Stimulated Raman Scattering Microscopy

An integrated laser system (picoEMERALD S, Applied Physics & Electronics, Inc.) was employed to generate two synchronized laser beams at 80 MHz repetition rate. A Stokes beam (1031.4 nm, 2 ps pulse width) was intensity modulated by an electro optic-modulator (EOM) with >90% modulation depth, and a tunable pump beam [700–960 nm, 2 ps pulse width, <1 nm (~10 cm^{-1}) spectral bandwidth] was produced by a built-in optical parametric oscillator (OPO). For SRS measurements, the Stokes beam was modulated with a 20 MHz EOM. The pump and Stokes beams were spatially and temporally overlapped via a series of dichroic mirrors and a delay stage inside the laser system, paired to an inverted laser-scanning microscope (Leica TCS SP8, Leica Microsystems), where the beams were focused onto the sample by a 40× objective (HC PL IRAPO 40×, N.A. 1.10 water immersion lens). Forward scattered light was collected by a S1 N.A. 1.4 condenser lens (Leica Microsystems). The Stokes light was removed and the pump beam intensity measured by a silicon photodiode connected to a lock-in amplifier (Applied Physics & Electronics, Inc.). The lock-in amplifier signal was fed into the Leica Microsystems SP8 microscope. The laser powers measured after the objective lens were in the range 10–30 mW for the pump beam only, 10–50 mW for the Stokes beam only and both synchronized beams at 20–70 mW. SRS images were acquired with a 9.75 μs pixel dwell time over a 512 × 512 frame at 12-bit image depth and recorded using Leica application suite (LAS X) software. The spatial resolution of the system is ~450 nm (pump wavelength = 792 nm). Polystyrene beads (~1 μm) were used to calibrate the multimodal setup through the detection of SRS signal at 3050 cm^{-1}. All images were captured using the aforementioned custom-built multi- photon confocal microscope at the University of Strathclyde.

SRS imaging and spectral phasor analysis. Harvested SK-BR-3 cells were seeded with a density of 0.3×10^6 cells onto high precision coverslips (#1.5H thickness, 22 × 22 mm, Thorlabs) in a six-well culture dish (Costar) with 2 mL of culture medium and incubated at 37 °C and 5% CO^2 for 24 h prior to the cell synchronization. Following a double thymidine block, SK-BR-3 cells were washed with PBS (2 × 2 mL) and fixed in 4% paraformaldehyde (2 mL for 15 min) at key time points (5–9 h) during mitotic entry. Following fixation, the coverslips were washed with PBS (2 × 2 mL) and mounted on glass microscope slides with a PBS boundary between the glass layers using a method as previously described in *Fu et al.*[2] For cell imaging, a typical field-of-view was between 30–100 μm^2 containing a maximum of 1–3 cells. A wavelength scanning experiment was performed by tuning the pump laser wavelength at increments of 0.4 nm (~6 cm^{-1}) across the range 2800–3050 cm^{-1} to collect a hyperspectral stack of 40 SRS images.

1.3 Fluorescence imaging

Fixed SK-BR-3 cells were treated with DAPI (1 μM, 10 mins). The cells were mounted in PBS and affixed onto a microscope slide as implemented for SRS imaging. Fluorescence images were acquired using a Leica Microsystems SP8 confocal microscope equipped with a 40× NA 1.10 water immersion objective lens. DAPI signal was detected λex= 405 nm, λem = 410-550 nm (Figure 2). False-color assignments were added on ImageJ.

1.4 Data Processing

SRS Images. False-color assignments, scale bars, and image overlays were added using ImageJ software. Consistent brightness/contrast settings were applied when comparing average intensity projections in all figures.

Spectral Phasor Analysis. The hyperspectral SRS dataset was acquired across the range 2800–3050 cm^{-1} using a 0.4 nm re-tune in the pump laser wavelength (40 images) which was imported into Image J as a stack. The 3D dataset was transformed into a 2D spectral phasor plot based on a Fourier transform[3] that considers the SRS spectrum at individual pixels for N sampled data points (N= 40 images). The equation also considers the spectral phasor order, k, (1st order) and the spectral range, Δ,. 250 cm^{-1}. These values remain consistent throughout the dataset acquisition for every spectral phasor plot that is presented in this work, and therefore, for simplicity, 1, 2, ..., 40 and N (40) replace the actual wavenumbers and the spectral range. The three dimensional spectral dataset is then projected in a 2D Fourier space with each point (referred to a spectral phasor) determined by the coordinates (u,v) therefore representing a single pixel (and hence spectrum) within the data stack.

$$u = \frac{\sum_{n=1}^{N} S(\lambda_n)\cos({}^{2\pi k\lambda_n}/_{\Delta})}{\sum_{n=1}^{N} S(\lambda_n)} \qquad v = \frac{\sum_{n=1}^{N} S(\lambda_n)\sin({}^{2\pi k\lambda_n}/_{\Delta})}{\sum_{n=1}^{N} S(\lambda_n)}$$

where $S(\lambda 1), S(\lambda 2), \ldots, S(\lambda N)$ is the SRS spectrum at individual pixels for N sampled spectral points ($\lambda 1, \lambda 2, \ldots, \lambda N$). k and Δ represent phasor order and spectral span, respectively[3].

All images were acquired as 512×512 pixels, therefore, the phasor plot contains 262, 144 phasor points which are displayed as a 2D plot, where the pseudo-color is used to represent the density of phasors at any given point in the Fourier space; pixels in the dataset that have the same SRS spectrum will therefore appear as a warmer color on the intensity scale of the LUT for the spectral phasor plot. We have cropped the spectral phasor plots to facilitate the visualization of the plot. Segmentation of the phasor plot was performed manually using regions-of-interest to create images of distinct cellular locations as described in Figure 1. The mean area of the segmented nuclei and cytoplasmic regions were determined by using the Analysis tool on ImageJ.

Statistical Analysis. Statistical analyses and data plotting were performed using GraphPad PRISM software v9.5.1 (GraphPad Software Inc., San Diego, CA, USA).

2. Supplementary figures

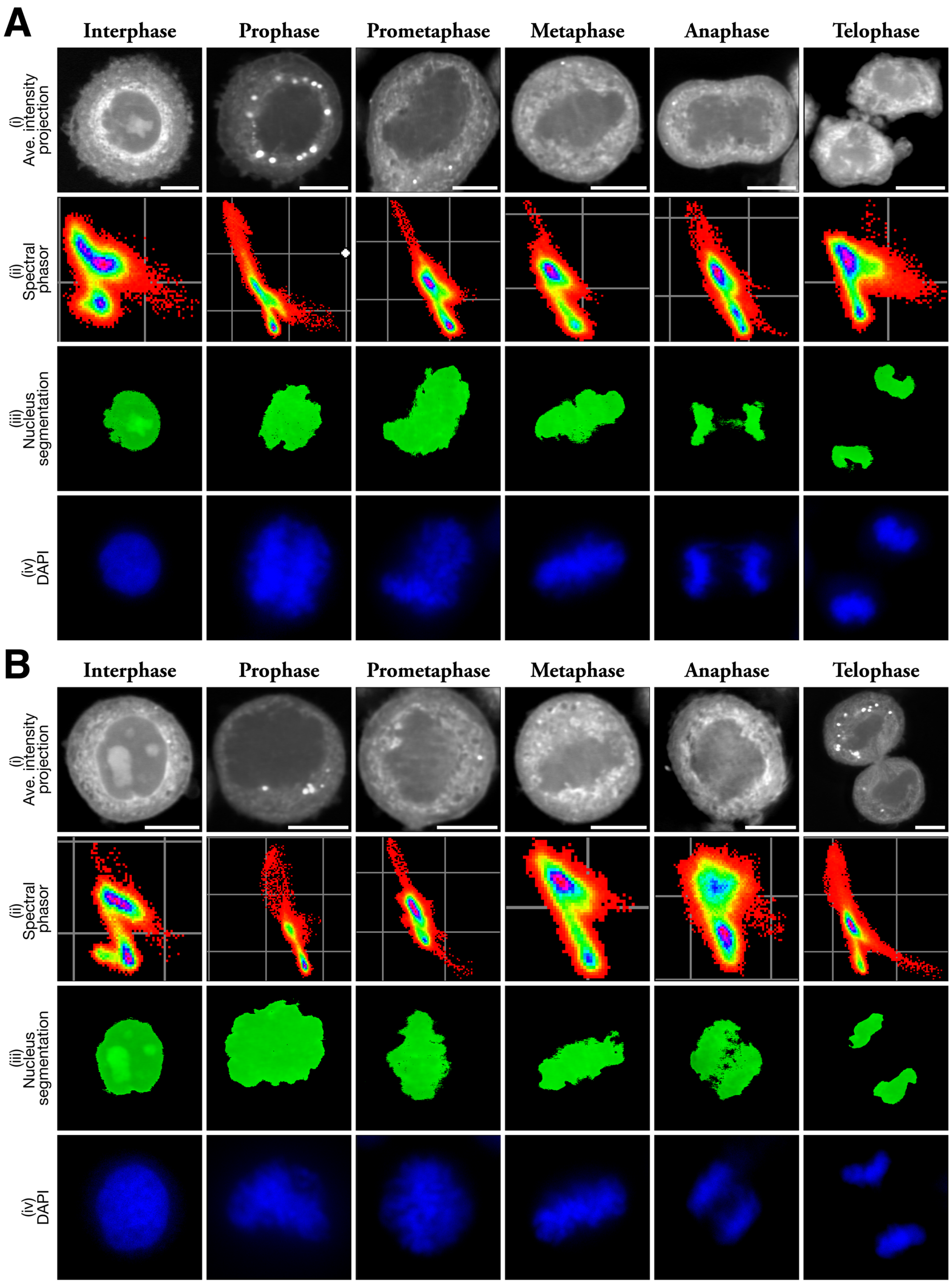

Figure S1 Hyperspectral profiling of mitotic cells using phasor analysis. Biological replicates, BR2 (A) & BR3 (B) were imaged at each of the five phases of mitosis and an interphase model for comparison. SRS images were acquired across a range of wavenumbers (2800–3050 cm^{-1}) and from this stack an average intensity projection (i) was generated (scale bar: 10 μm). Spectral phasor plots (ii) were produced from each mitotic phase and segmentation of the nucleus (iii). Fluorescence imaging of DNA contrast agent (DAPI) (iv).

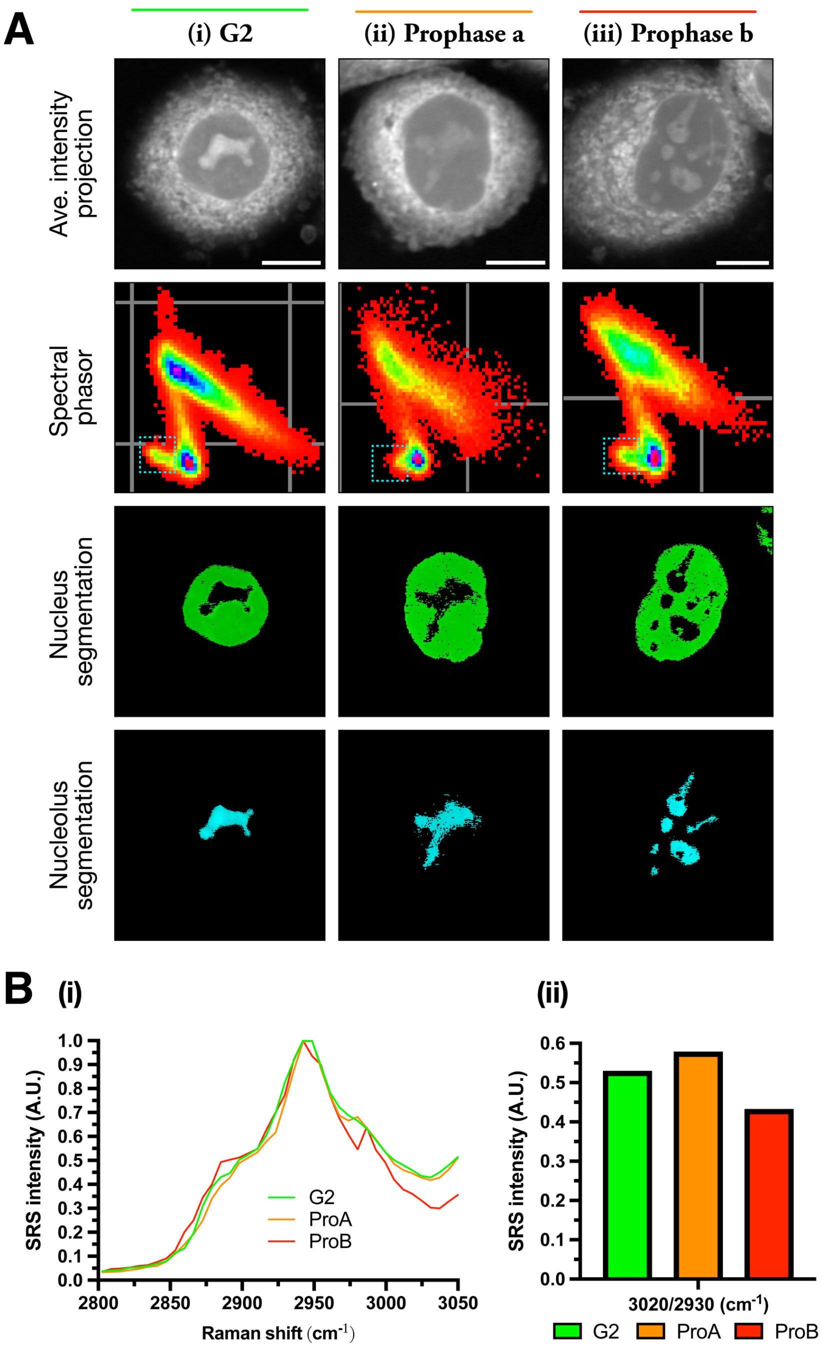

Figure S2 SRS imaging of nucleolar disassembly at the beginning of mitosis. SRS images of SK-BR-3 cell model at different steps of ribosome biogenesis. Adapted from a schematic illustration presented in Hernandez-Verdun[4] different nucleoli morphologies are captured of the ordered release of nucleolar complexes following the repression of RNA transcription. (A) G2 (i), prophase a (ii) when processing is disrupted and prophase b (iii) when transcription is extinct. SRS images were acquired across a range of wavenumbers (2800–3050 cm^{-1}) and from this stack an average intensity projection was generated (scale bar: 10 μm), corresponding phasor plots were produced from each mitotic phase and segmentation of the nucleus and nucleoli. Nucleolar ROI (cyan). (B) Spectra associated with signal from the nucleoli (i) of the SRS image were extracted by phasor analysis. Ratiometric analysis of the intensity signal (ii) at 3020/2930 cm^{-1} (RNA/ CH_3) from the nucleoli at different stages of RNA processing. Graphs were normalized between 0-1.

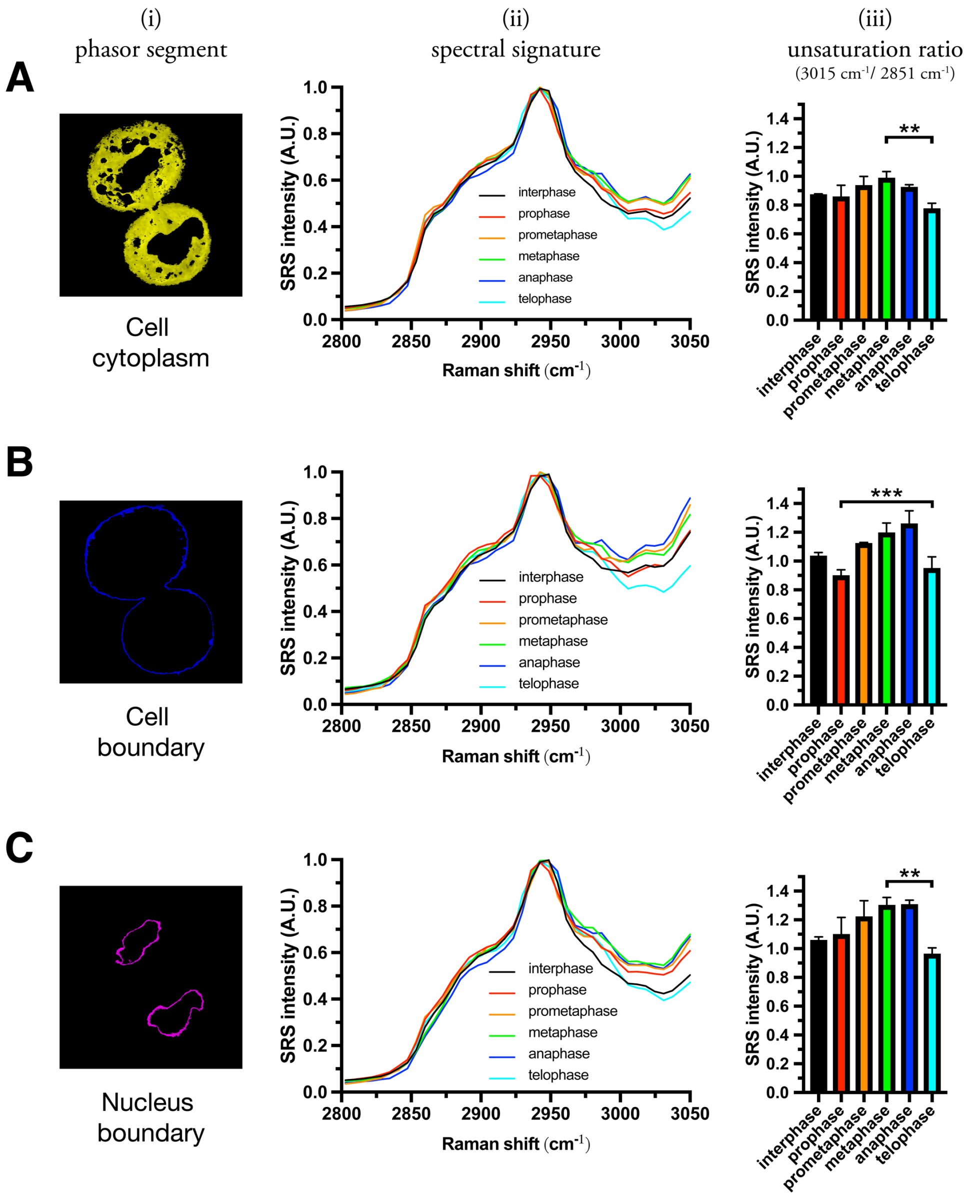

Figure S3 Investigating membrane associated regions of mitotic cells by phasor analysis. SRS images of SK-BR-3 cell model at different cell cycle phases were collected across a range of wavenumbers (2800–3050 cm^{-1}) and from this stack the cytoplasm (A), cell boundary (B) and nuclear boundary (C) were assessed by phasor segmentation (i). The average cell spectrum across the HWN is plotted from each of the segmented regions of interest (ii). The ratio of the intensity of the peaks at 3015 cm^{-1}/2851 cm^{-1} is plotted as a measurement of unsaturation in these regions of the cell (iii). Three replicate analyses were each cell cycle phase. Data represent the mean ratio ± S.D. A one-way Anova test with Tukey post-hoc analysis was performed $^{**}P \leq 0.01$, $^{***}P \leq 0.001$.

3. References

(1) Banfalvi, G. *Cell Cycle Syncronization: Methods in Molecular Biology*, 2nd edition.; HUMANA Press, 2018.

(2) Fu, D.; Zhou, J.; Zhu, W. S.; Manley, P. W.; Wang, Y. K.; Hood, T.; Wylie, A.; Xie, X. S. Imaging the Intracellular Distribution of Tyrosine Kinase Inhibitors in Living Cells with Quantitative Hyperspectral Stimulated Raman Scattering. *Nature Chem.* **2014**, *6* (7), 614–622.

(3) Fu, D.; Xie, X. S. Reliable Cell Segmentation Based on Spectral Phasor Analysis of Hyperspectral Stimulated Raman Scattering Imaging Data. *Anal. Chem.* **2014**, *86* (9), 4115–4119.

(4) Hernandez-Verdun, D. Assembly and Disassembly of the Nucleolus during the Cell Cycle. *Nucleus* **2011**, *2* (3), 189–194.
